# SeptiFast versus blood culture in clinical routine – A report on 3 years experience

**DOI:** 10.1007/s00508-017-1181-3

**Published:** 2017-02-27

**Authors:** Florian Korber, Iris Zeller, Michaela Grünstäudl, Birgit Willinger, Petra Apfalter, Alexander M. Hirschl, Athanasios Makristathis

**Affiliations:** 1 Division of Clinical Microbiology, Department of Laboratory Medicine, Medical University Vienna, Währinger Gürtel 18-20, 1090 Vienna, Austria; 2 Praxis Dr. med. Norbert Haßfurther, Launsbach, Germany; 3 Krankenhaus der Elisabethinen Linz, Linz, Austria

**Keywords:** SeptiFast, Blood culture, Sepsis, Routine use, Diagnostics

## Abstract

**Background:**

In recent years a multiplex real-time PCR (SeptiFast) has been introduced, allowing detection of 25 common blood pathogens considerably faster than conventional blood culture.

**Methods:**

SeptiFast was applied routinely in addition to blood culture in cases of critically ill patients with fever and other signs of severe systemic infections. In this study data of 470 episodes were retrospectively analysed to assess the impact of various parameters, such as clinical indications, assigning ward and antimicrobial treatment on test outcome using a multivariate logistic model.

**Results:**

After exclusion of microorganisms classified as contaminants, the concordance between SeptiFast and blood culture was 85.5%. SeptiFast detected 98 out of 120, while blood culture merely found 63 out of 120 potential pathogens. In comparison to blood culture, SeptiFast showed considerably higher positivity rates in sepsis, pneumonia and febrile immunosuppression and a lower rate in endocarditis. The highest positivity and concordance between tests was shown in patients from the emergency room (*P* = 0.007).

**Conclusions:**

The results obtained in this study are similar to those from prospective settings confirming the robustness of the SeptiFast assay in routine use. Our data suggest that SeptiFast is a valuable add-on to blood culture and may increase the diagnostic efficiency of a microbiological laboratory.

## Introduction

In 2003 sepsis was estimated to be the third most common cause of death overall, and its incidence is still rising [1–4]. Fatality rates range from 10% for children to 38.4% for elderly people, and sepsis accounts for approximately 30% of healthcare costs in intensive care units (ICU) [5].

Blood culture (BC) still is the gold standard for microbiological sepsis diagnosis [6–8]; however, this method lacks sensitivity, especially for slowly growing or fastidious microorganisms and fungi. Substantial time delays due to impaired growth kinetics of blood microorganisms after antimicrobial treatment, low initial pathogen load and volume of specimen inoculated, are frequently observed [9]. Numerous non-infectious conditions can mimic sepsis, and the rapid detection of the causative pathogen is of particular importance since therapy and outcome differ greatly between patients with sepsis and those with non-infectious conditions. While timely administration of adequate antimicrobial therapy is of paramount importance for the survival of patients with sepsis, inappropriate antibiotic therapy is not only associated with reduced survival rates but also with increasing antibiotic resistance, toxicity, costs and longer hospital stays [10–20]. To meet the need for faster microbiological diagnostics, other methods have been introduced into routine clinical laboratories, including improved culture media, automated blood culture systems, and molecular tests [21, 22]. SeptiFast (SF; Roche Diagnostics, Mannheim, Germany) was the first real-time multiplex polymerase chain reaction (PCR) test for whole blood samples. It covers the 19 most frequent bacterial and 6 most common fungal pathogens of bloodstream infections. Originally developed and applied for patients with sepsis [23–34], it is currently also used for other indications, e. g. patients with febrile neutropenia, endocarditis, fever of unknown origin (FUO) and invasive aspergillosis [22, 29, 35–38]. With a 5-h run time, SF is considerably faster than conventional BC testing [24, 39, 40]. The SF test was introduced into the daily clinical routine of our institution as an add-on to the conventional laboratory sepsis diagnostics. During the first 3 years paired BC and SF testing was implemented because clinical data regarding the performance of SF were sparse at that time. In the present study, we retrospectively analysed SF and BC results of this period from patients with sepsis and/or evidence of other severe systemic infectious diseases in association with parameters, such as clinical indications, ward and antimicrobial therapy. For a subset of ICU patients the impact of positive results on total hospital as well as ICU stay and mortality rate was also investigated.

## Patients, materials and methods

### Data acquisition

This study was conducted as a retrospective observational study and was approved by the institutional review board of the Medical University Vienna and performed in accordance with the ethical standards as laid down in the 1964 Declaration of Helsinki and its later amendments. For this type of study informed consent is not required. Data were collected from cases analysed at the Division of Clinical Microbiology of Vienna’s General Hospital during a 3-year period (2007–2010). Patients were included in the study only if both BC and SF analyses were performed; specimens for BC and SF testing had to be taken during the same blood draw or within 24 h of each other. All clinical data were extracted from the information provided on test request forms and the patient medical records. Indications were sepsis, endocarditis, FUO, pneumonia and immunosuppression (e. g. due to solid organ or bone marrow transplantation or hematological malignancies). Test results from patients’ other microbiological samples (OMS; other blood cultures, specimens from central venous catheters, the lower respiratory tract, surgical sites, intra-abdominal swabs, urine, other body fluids and tissue specimens) were taken into consideration only if collected within 2 days of the blood draw for SF/BC. Antibiotic treatment was considered relevant if it had been administered within 24 h before testing. Clinical wards submitting the samples were internal, surgical, ICUs (surgical and internal), the emergency department, and outpatient units.

If common contaminants of the resident skin flora, e. g. coagulase-negative staphylococci (CoNS) were detected by BC, they were regarded as relevant microorganisms only after the consideration of certain clinical conditions (e.g. endocarditis and central venous catheter associated infections) in conjunction with laboratory data (time to positivity of the BC ≤ 24 h and growth of the same microorganism in both aerobic and anaerobic bottles in the case of a single BC set, growth in multiple BC sets from the same blood draw, or growth in OMS e. g. culture of the central venous catheter).

When detected by the SF system software, CoNS were considered to be relevant if the above-named clinical conditions were met. In some cases, CoNS detection was associated with a low peak during melting curve analysis following PCR, which was not identified by the software. In these cases, after manual interpretation of the data, the CoNS were considered relevant if they were also identified by BC and met the other criteria described.

### Laboratory procedures

The BC samples each consisted of an aerobic and anaerobic bottle (usually 1–3 sets of the same episode). The BC samples were incubated at 36.5–37 °C for up to 7 days in the semi-automated continuous monitoring BC system BacT/ALERT 3D (BioMérieux, Marcy l’Etoile, France). Gram staining and subcultures on solid media were performed from positive BCs. Isolates were then identified via standard microbiological methods, Vitek II (BioMerieux), or matrix-assisted laser desorption ionization-time of flight mass spectrometry (MALDI-TOF MS; Microflex; Bruker Daltonics, Bremen, Germany). The SF testing was performed as suggested by the manufacturer. All results were checked manually in the software.

### Statistical procedures

A multivariate logistic regression was used to analyse the association of the SF and BC positivity rates with predicting test variables (clinical indications, assigning ward and antimicrobial treatment) after cases of contamination were removed. The comparison of SF+/BC− vs. SF−/BC+ for each of the categories of a test variable was carried out by binomial tests. A possible impact on the distribution of SF+ vs. SF− test results was compared for each category using a χ^2^-test. To analyse the impact of positive SF results on the length of ICU stay, total hospital stay, and mortality, ICU patients with single (SF+/BC−) and double positive (SF+/BC+) test results were matched with double negative (SF−/BC−) cases with the same gender, age and clinical ward. Matching was performed using a log-rank test. Patients who died during the hospital stay were counted as deceased and evaluated separately using a χ^2^-test. For all tests an alpha value of 0.05 was chosen as significance level.

## Results

We derived 470 paired BC and SF samples from 398 patients. Only one (paired) sample was submitted from 345 patients, whereas multiple sample pairs were submitted from 53 patients. Multiple pairs from the same patient came from independent episodes; therefore, the samples were treated as independent samples for our study. In 419 cases, SF and BC samples were collected from the same blood draw, in 51 cases from different blood draws (within 24 h). Of the 470 febrile episodes analysed, the clinical indications for the blood draw were sepsis in 190, endocarditis in 46, FUO in 56, pneumonia in 97, immunosuppression in 93 (67 with status post-organ transplantation, and 29 with hematological malignancies) and for 16 cases the exact indication could not be reconstructed. In 110 cases more than one clinical indication for testing was given. In 147 (31.3%) out of 470 cases at least 1 of the tests was positive (Table 1). Overall, SF was positive in 125 (26.6%), and BC in 71 (15.1%) cases. Both tests were negative in 323 (68.7%), and both were positive in 49 (10.4%) cases. After exclusion of contaminants, a total of 427 cases remained in the study. Of these, 75.6% yielded double negative results, and 104 (24.4%) positive results, including 44 (10.3%) double positive results. For 2 of the double positives, microorganisms identified by SF and BC differed from each other. In one case, SF revealed *Enterobacter spp*. and *Klebsiella spp*., while BC identified *Comamonas testosteroni*, which is not part of the SF panel. In the other case SF detected *Candida albicans* (which was also found in OMS), whereas BC identified *Klebsiella pneumoniae, Enterococcus faecalis*, and CoNS. The SF and BC results agreed in 365 of the 427 (85.5%) cases.

**Table 1:** Overview of positive test results

Category	Positive samples (% of total 470)	Positive samples with relevant microorganisms (% of total 427)	Microorganisms (% of total)	Relevant microorganisms (% of total)
SF+/BC−	76 (16.2)	44 (10.3)	105 (58.0)	57 (47.5)
SF−/BC+	22 (4.7)	16 (3.7)	31 (17.1)	22 (18.3)
SF+/BC+	49 (10.4)	44 (10.3)	45 (24.9)	41 (34.2)
Overall	147 (31.3)	104 (24.4)	181 (100)	120 (100)

*SF* SeptiFast, *BC* blood culture

Overall, SF detected a total of 150 microorganisms, 98 of which were considered relevant (65.3%); the vast majority of positive but irrelevant results were CoNS detected only by manual interpretation of SF results. BC detected 76 microorganisms, and 63 were classified as relevant (82.9%). Thus, of a total of 120 relevant microorganisms identified, 81.7% were detected by SF, and 52.5% by BC. In 6 out of 16 cases (6/22 microorganisms, Table 2) in which a potential pathogen was solely detected by BC, the microorganism was not included in the SF master detection list. In the 44 cases where relevant pathogens were only detected by SF, 57 microorganisms were detected (53 common sepsis pathogens and 4 common contaminants), which were considered as relevant. Of the 53 common sepsis pathogens 16 were also detected in at least 1 OMS (Table 2).

**Table 2:** Overall distribution of detected microorganisms

Microorganisms		Total	SF+	BC+	SF+/BC- (OMS+)	SF-/BC+	SF+/BC+
Gram+	*Staphylococcus aureus*	18	17	10	8 (2)	1	9
	CoNS	9	6	8	1	3	5
	*Streptococcus spp*	4	4	1	3 (1)	0	1
	*Streptococcus pneumoniae*	12	12	3	9 (2)	0	3
	*Enterococcus faecium*	8	6	2	6 (5)	2	0
	*Enterococcus faecalis*	3	2	2	1	1	1
Gram−	*Escherichia coli*	19	16	14	5 (1)	3	11
	*Klebsiella pneumoniae*/*Klebsiella oxytoca*	9	7	5	4	2	3
	*Serratia marcescens*	1	1	1	0	0	1
	*Enterobacter cloacae*/*Enterobacter aerogenes*	7	7	1	6	0	1
	*Proteus mirabilis*	0	0	0	0	0	0
	*Pseudomonas aeruginosa*	7	5	4	3	2	2
	*Acinetobacter baumannii*	1	0	1	0	1	0
	*Stenotrophomonas maltophilia*	1	0	1	0	1	0
Fungi	*Candida albicans*	9	9	3	6 (2)	0	3
	*Candida tropicalis*	2	2	0	2 (1)	0	0
	*Candida parapsilosis*	0	0	0	0	0	0
	*Candida krusei*	1	1	1	0	0	1
	*Candida glabrata*	0	0	0	0	0	0
	*Aspergillus fumigatus*	3	3	0	3 (2)	0	0
Cont.	CoNS	56	48	12	44 (1)	8	4
	*Streptococcus spp*	4	4	0	4	0	0
	Propionibacterium acnes*	**1**	**0**	**1**	**0**	**1**	**0**
Other	Comamonas testosteroni*	**1**	**0**	**1**	**0**	**1**	**0**
	Bacteroides spp*	**2**	**0**	**2**	**0**	**2**	**0**
	Granulicatella spp*	**1**	**0**	**1**	**0**	**1**	**0**
	Listeria monocytogenes*	**1**	**0**	**1**	**0**	**1**	**0**
	Prevotella spp*	**1**	**0**	**1**	**0**	**1**	**0**

*SF* SeptiFast, *BC* blood culture, *OMS+* SeptiFast result confirmed by other microbiological samples, *Gram+* relevant gram-positive bacteria, *Gram−* relevant gram-negative bacteria, *Cont.* contaminants, *CoNS* coagulase-negative staphylococci

* microorganisms not included in the SeptiFast master detection list

When compared with BC, SF was more likely to identify *Staphylococcus aureus* (17/18 vs. 10/18), *Streptococcus pneumoniae* (12/12 vs. 3/12), *Enterococcus faecium* (6/8 vs. 2/8), *Enterobacter spp*. (7/7 vs. 1/7), *Candida spp*. (12/12 vs. 4/12) and *Aspergillus fumigatus* (3/3 vs. 0/3) (Table 2). In 5/8 *S. aureus*, 8/9 *S. pneumonia*e, 4/6 *E. faecium*, 4/6 *Enterobacter spp*., 6/8 *Candida spp*., and 3/3 *A. fumigatus* cases found only by SF, antimicrobial agents had been administered prior to the blood draws. Antimicrobial treatment was carried out in 31 of the 44 SF+/BC− cases with relevant pathogens. Overall, of the 427 uncontaminated sample pairs, 276 (64.6%) were derived from patients under antimicrobial therapy. For treated patients the number of SF+/BC− outcomes was significantly higher than that of SF−/BC+ cases (*P* < 0.001); this was not found in untreated patients. The ratio of SF+ to SF− test results did not differ considerably between the categories therapy and no therapy (Table 3).

**Table 3:** Statistical analysis of differences per test variable in samples with relevant pathogens

Test variable	*P* (SF+/BC− vs. SF−/BC+)	*P* (SF+ vs. SF−)
Sepsis	<0.001 (+)	<0.001 (+)
Endocarditis	0.013 (−)	0.568
FUO	0.125	0.011 (−)
Pneumonia	<0.001 (+)	0.202
Immunosuppression	<0.001 (+)	0.179
Internal medicine	0.004 (+)	0.003 (−)
Surgery	0.009 (+)	0.089
ICU	0.006 (+)	0.002 (+)
Emergency	<0.001 (+)	0.002 (+)
Outpatient	n.a.	0.170
Therapy	<0.001 (+)	0.779
No therapy	0.089	0.779

*P P*-value, *SF* SeptiFast, *BC* blood culture, (+) significantly higher, (−) significantly lower, *n.a.* not available, *FUO* fever of unknown origin, *ICU* intensive care unit

**Fig. 1:**
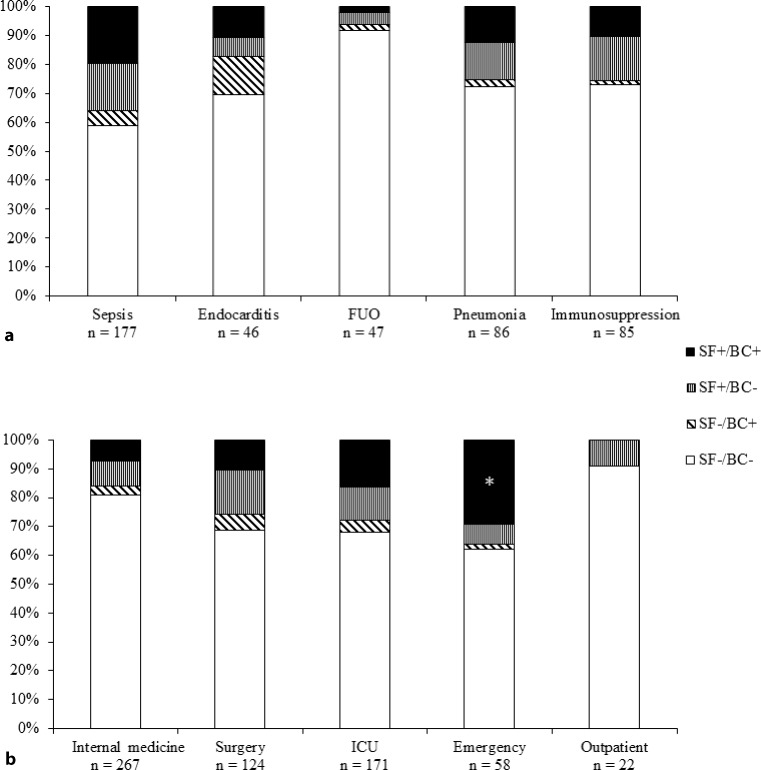
Distribution of SeptiFast (*SF*) and blood culture (*BC*) test results with respect to the clinical indications (**a**) and ward (**b**) after exclusion of cases with microorganisms classified as contaminants. Intensive care unit (*ICU*) patients are also included under internal medicine and surgery. The majority of emergency department patients are also included in the internal medicine group, as most of these patients were transferred to an internal medical ward. (*) the rate of SF+/BC+ results for samples submitted from the emergency department was significantly higher than that of any of the other wards, *FUO* fever of unknown origin

Fig. 1 shows the distribution of SF and BC results of uncontaminated sample pairs with respect to clinical indications and assigning ward. With respect to clinical indications, the ratio of SF+ to SF− outcomes was significantly higher for sepsis and lower for FUO in comparison to the other categories (Table 3). Furthermore, the number of SF+/BC− cases was significantly higher than that of SF−/BC+ outcomes for the categories sepsis, pneumonia, and immunosuppression, but lower for endocarditis (Table 3). The ratios of treated to untreated patients were comparable between the different categories e. g. 73.7% for sepsis and 69.6% for endocarditis.

Regarding the variable “ward”, the number of SF+/BC− test results was significantly higher than that of SF−/BC+ outcomes (Table 3) in each but the outpatient wards, where the low number of specimens did not allow for statistical analysis. The ratios of SF+ to SF− test results were significantly higher for specimens submitted by the emergency department and ICUs, and significantly lower for those from internal medical wards (Table 3). The highest overall positivity (23/58; 39.7%) and highest rate of SF+/BC+ outcomes (18/58, 31.0%; 100% concordance) but at the same time, with the exception of outpatient units, the lowest ratio of treated to untreated patients by far (35%), was shown for cases from the emergency department. Multivariate logistic regression analysis revealed that the percentage of SF+/BC+ cases for the emergency department was significantly higher (*P* = 0.007) than that for any of the categories irrespective of the test variable (Fig. 1).

The 17 SF+/BC+ and 19 SF+/BC− cases from ICUs were matched with equal numbers of SF−/BC− ICU cases. Age, gender and clinical ward were used as matching criteria, the clinical diagnosis and antibiotic therapy were also comparable between the groups. When analysing the length of ICU and total hospital stay no significant differences were found between SF+/BC+ and SF+/BC− vs. SF−/BC− cases. Deceased patients were excluded from this analysis. A total of 15/36 (41.7%) patients died in the SF+ vs. 9/36 (25%) in the SF− cases, 7/19 (36.8%) patients died in the SF+/BC− vs. only 2/19 (10.5%) in the SF−/BC− control group and 8/17 (47.1%) patients died in the SF+/BC+ vs. 7/17 (41.2%) in the control group. These differences were not statistically significant.

## Discussion

The present study on patients with sepsis or evidence of other serious systemic infectious diseases aimed at the retrospective assessment of the usage of SF in a routine setting. A particular focus was set on the association of clinical factors and the assigning ward with the rate of pathogen identification by SF in comparison to BC. This knowledge might contribute to a more efficient routine application of this molecular test.

In this retrospective analysis overall positivity rates of SF and BC and concordance between tests were similar to data published in prospective studies [27, 34]. Thus, the positivity rates were 26.6% for SF and 15.1% for BC. After exclusion of contaminants, the rate of false negative cases in SF was 3.7%, the test agreement rate 85.5%, and the percentage of cases of relevant pathogens solely detected by SF 10.3%. While the rates of relevant pathogens found by SF (82%) and BC (53%) in the present study were comparable to other studies, the rate of contaminants detected by SF was considerably higher than reported previously [22, 28, 31, 34, 39]. The SF cut-off value for common contaminants is set higher than that for other bacteria to avoid false positive findings due to bacterial skin flora contamination. Thus, low melting curve peaks in the spectrum of common contaminants are normally not identified by the software, and the respective samples are regarded as negatives; however, according to our routine laboratory guidelines, SF results are to be interpreted manually. This is due to our observation that, particularly in the gram-positive spectrum, pathogens also detected by BC may not be identified by the SF software due to low melting curve peaks. Low melting curve peaks corresponding to the spectrum of CoNS are routinely communicated to the clinician as possible contamination. In a considerable number of BC negative cases relevant pathogens could only be detected by SF, as also described in several prospective studies [22–30, 32–34, 36, 38]. Moreover, with the exception of FUO and endocarditis, in other categories the number of SF+/BC− cases was significantly higher than that of SF−/BC+ cases. The fact that SF may underperform for patients suffering from endocarditis has been demonstrated by others [35]. This may be due to several reasons: the small numbers of pathogens circulating in blood, the fact that some endocarditis-relevant pathogens (e. g. those of the HACEK group: *Haemophilus spp*., *Aggregatibacter actinomycetemcomitans, Cardiobacterium hominis, Eikenella corrodens, Kingella kingae*) are not included in the SF panel, or the high cut-off value for CoNS; however, the last two reasons do not apply for the present study. Nevertheless, Mencacci et al. suggest that SF may be a useful tool for the detection of endocarditis pathogens in patients undergoing antimicrobial therapy [41].

In agreement with previous studies [24, 27–29, 34, 38], *S. aureus, S. pneumoniae, E. faecium, Enterobacter spp*., and fungi were detected more frequently by SF than BC. In the great majority of SF+/BC− cases with these pathogens the patients were under antimicrobial therapy. This suggests that treatment may, at least in part, be responsible for this discrepancy. The potential role of antimicrobial therapy is also supported by the significantly higher number of SF+/BC− cases, compared to SF−/BC+ cases, in treated patients; however, the difference in the rate of SF+/BC− test results between the treated and the untreated group was not significant. In this respect our data are in line with studies [22, 38] that demonstrated a higher rate of positivity of SF for the treated group with the difference, however, not reaching statistical significance. A significant difference has been reported in other studies [42–44].

In samples submitted from the emergency department we observed a significantly higher rate of SF+/BC+ results compared to any other ward. We also noted that for all SF+/BC+ cases in this group the SF and BC results were in agreement. This finding may be due to the following: patients delivered to the emergency unit have more apparent signs and symptoms of sepsis than those admitted to other wards (e. g. ICUs); therefore, the decision to perform BC and SF as specific diagnostic measures may be more obvious. Additionally, these patients usually have not received antimicrobial therapy, which might contribute to the high overall positivity and agreement between both tests. Thus, emergency room patients may benefit most from the rapid results obtained by SF testing rather than higher test positivity in comparison to BC. Our findings are in accordance with those by Avolio et al. [23] but diverge from those reported by Tsalik et al. [31].

Lehmann et al. [27] demonstrated significantly longer hospital stays (38 vs. 23 days), and a higher rate of mortality (66% vs. 33%) for patients with SF+/BC+ vs. SF−/BC− test results. The lack of considerable differences in the length of ICU and total hospital stays between ICU patients with SF+/BC− or SF+/BC+ test results and matched SF−/BC− controls in our study may be explained by the fact that patients with positive test results, while being more ill, are given a more targeted antibiotic therapy. The higher rate of mortality for SF+ than SF− patients may indicate that a positive PCR result is likely to reflect a severe life-threatening medical condition; however, these findings need to be considered with appropriate caution considering the small subset of data analysed.

Since the introduction of the SF assay several prospective but only few retrospective studies evaluated the benefits and shortcomings of this method [45–47]. To our knowledge, this study is so far the only one retrospectively analysing the association of the test outcome with important factors such as the indications for testing, assigning ward and antimicrobial therapy in comparison to BC; however, even though retrospective studies may reflect clinical practice more accurately than prospective controlled trials, they are more prone to bias. In this context, it needs to be noted that the decision for SF testing was solely taken by the responsible clinician. Thus, a selection bias is likely to have influenced the rate and types of pathogens found.

SF showed a low rate of false negative results and, due to its fast turnaround time, proved to be a valuable diagnostic add-on to BC. The highest positivity and concordance between tests was shown in emergency room patients. Overall, in cases of suspected sepsis, pneumonia, or in immunocompromised patients with febrile episodes, SF exhibited considerably higher positivity rates and enabled the rapid detection of clinically important pathogens not identified in BC; however, the clinical relevance and significance of SF+/BC− results need to be interpreted with caution and closely focused prospective studies are needed to clarify this important issue:
